# What happens to lost nets: a multi-country analysis of reasons for LLIN attrition using 14 household surveys in four countries

**DOI:** 10.1186/1475-2875-13-464

**Published:** 2014-11-27

**Authors:** Hannah Koenker, Albert Kilian, Celine Zegers de Beyl, Emmanuel O Onyefunafoa, Richmond A Selby, Tarekegn Abeku, Megan Fotheringham, Matthew Lynch

**Affiliations:** Johns Hopkins Bloomberg School of Public Health Center for Communication Programs, Baltimore, MD USA; Tropical Health LLP, Montagut, Girona, Spain; Malaria Consortium, London, UK; Malaria Consortium, Abuja, Nigeria; Malaria Consortium, Kampala, Uganda; United States Agency for International Development, Washington, DC USA

## Abstract

**Background:**

While significant focus has been given to net distribution, little is known about what is done with nets that leave a household, either to be used by others or when they are discarded. To better understand the magnitude of sharing LLIN between households and patterns of discarding LLIN, the present study pools data from 14 post-campaign surveys to draw larger conclusions about the fate of nets that leave households.

**Methods:**

Data from 14 sub-national post-campaign surveys conducted in Ghana, Senegal, Nigeria (10 states), and Uganda between 2009 and 2012 were pooled. Survey design and data collection methods were similar across surveys. The timing of surveys ranged from 2–16 months following their respective mass LLIN distributions.

**Results:**

Among the 14 surveys a total of 14,196 households reported owning 25,447 nets of any kind, of which 23,955 (94%) were LLINs. In addition, a total of 4,102 nets were reported to have left the households in the sample: 63% were discarded, and 34% were given away. Only 255 of the discarded nets were reported used for other purposes, representing less than 1% of the total sample of nets. The majority (62.5%) of nets given away were given to or taken by relatives, while 31.1% were given to non-relatives. Campaign nets were almost six times (OR 5.95, 4.25-8.32, p < 0.0001) more likely to be given away than non-campaign nets lost during the same period. Nets were primarily given away within the first few months after distribution. The overall rate of net redistribution was 5% of all nets.

**Discussion and conclusion:**

Intra-household re-allocation of nets does occur, but was sensitive to current household net ownership and the time elapsed since mass distribution. These factors can be addressed programmatically to further facilitate reallocation within a given community. Secondly, the overwhelming majority of nets were used for malaria prevention. Of the repurposed nets (<1% overall), the majority were already considered too torn, indicating they had already served out their useful life for malaria prevention. National programmes and donor agencies should remain confident that overall, their investments in LLIN are being appropriately used.

## Background

Long-lasting insecticidal nets (LLIN) are the main preventive tool against malaria, providing a reduction in malaria episodes of 50% [1]. The World Health Organization (WHO) recommends implementing universal coverage of LLIN for all populations at risk [2], and since 2004, over 800 million nets have been delivered to sub-Saharan Africa [3], primarily through mass campaigns, but also through antenatal care services, immunization clinics, and the retail sector. While significant focus has been given to net distribution, little is known about what is done with nets that leave a household, either to be used by others or when they are discarded.

The loss of nets from households, or net attrition, is important for two reasons. First, attrition is a significant component of calculating LLIN durability. Durability is calculated based on the direct observation of the number of nets given out originally that have survived to a certain time point, minus those that have been lost to follow-up and need to be accounted for in this denominator [4]. These lost nets are divided into two categories: a) those that are given away, sold, or stolen, but cannot be assessed and therefore are excluded from the denominator, and b) those that were thrown away, destroyed, or used for another purpose, which are included in the denominator. The decision to discard nets is, therefore, one of the principal drivers of calculating overall LLIN durability. Secondly, the percent of surviving nets is measured using information on the number and proportionate hole index of existing LLIN, to obtain the median net lifespan for a given crop of nets, and the importance of correctly doing so is well-described in the WHO Guidelines on measuring net durability [5]. Despite this, many recent durability studies [6–10] fail to include this measurement when they are calculating lifespans of LLIN in a given sample.

Household decisions around end of net life are highly subjective. Decision-making about when a net is no longer useful has been discussed in one study in Senegal [11], where respondents were asked hypothetical questions about when they would discard nets in varying degrees of disrepair, and what they would do with it. Most respondents stated that they would prefer to get a new net when possible rather than attempt to repair their nets when damaged. Batisso *et al.*[7] found in Ethiopia that the primary reason for non-use was that nets were considered too old or torn, although the condition of these ‘unusable nets’ was similar to other nets in use in the community: nets were considered old when they only had a few holes. One third of nets were discarded when they were just under a year old, but the physical condition of these nets was not reported. Reports from qualitative research in Madagascar on decisions to give up nets for recycling also shed light on the reasons why households might prefer to discard or keep old nets. These depended on whether the family felt they had sufficient nets to protect all family members, whether they had paid for the net or received it free, whether they were currently using it for an alternative purpose, among other reasons [12].

There is little information on the extent to which LLIN are shared between families and within communities, although recent unpublished data from a study on the effects of Hang Up activities in Uganda [13] indicate that nets that were given away were primarily given to family members who reside elsewhere, particularly students away at school. An older study in Tanzania recorded that between 6% and 20% of nets used the previous night were obtained as gifts from relatives or friends [14]. Sharing of LLIN is an important question in the context of recent community distribution strategies that may not target every household, for example school distribution strategies [15] or other strategies that may rely in part on households sharing nets with others that were not reached.

Lastly, there is evidence that LLIN are used for other purposes in some instances or in certain communities. While the overall percentage of nets that are used for purposes other than sleeping has been shown to be small [16], there are documented cases of nets being used for other purposes in coastal Kenya [10], for drying fish near Lake Victoria [17], for fishing in the Tamatave region of Madagascar (Andrea Brown & Mohamad Sy-Ar, personal communication), and for fishing in Lake Tanganyika [18]. Other studies [19–22] note that alternative use of LLINs occurs without being able to quantify the extent or nature of the practice.

To better understand the magnitude of sharing LLIN between households and patterns of discarding LLIN, and because the number of nets ‘lost’ in any given post-campaign survey is insufficient for individual analysis, the present study pools data from 14 post-campaign surveys to draw larger conclusions about the fate of nets that leave households.

## Methods

The post-campaign surveys were conducted to measure LLIN ownership and use following mass campaigns in four countries. The 14 subnational surveys were conducted in Ghana (Northern and Eastern regions), Senegal (single survey covering Kaffrine, Kaolack, Kolda, Sedhiou, Kedougou, and Tambacounda regions), Nigeria (Kano, Anambra, Sokoto, Niger, Ogun, Nasarawa, Katsina, Cross River, Enugu, and Lagos states), and Uganda (Western Uganda region) between 2009 and 2012. Survey design and data collection methods were similar across surveys, i.e. representative cross sectional household surveys with a two-stage cluster sampling design and a standard questionnaire. Analysis included 14,196 households and accounted for cluster survey design and sampling probabilities. The timing of surveys ranged from 2–16 months following their respective mass LLIN distributions.

**Figure 1:**
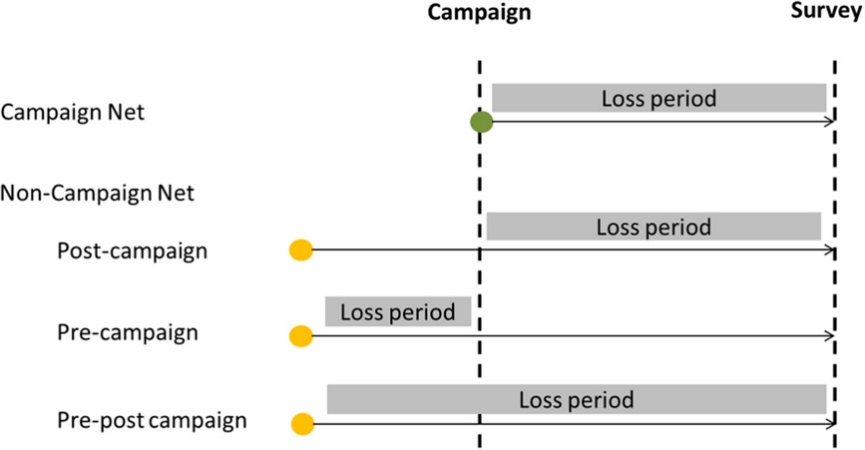
**Loss periods for campaign nets and non-campaigns nets.**

The data collection tool consisted of the standard MIS questionnaire with the basic household module and a household member and net roster [23]. Modules on the process of obtaining nets from the campaign and ownership of previous nets were added. Previously owned nets were divided into two broad categories, namely nets obtained from the campaign and those from other sources obtained before the campaign. As shown in Figure 1, any loss between the campaign and the survey was considered as “post-campaign” while a net owned before the campaign and lost within the 12 months preceding the campaign, i.e. no longer present at the time of the campaign, were considered as “pre-campaign” losses. In the Ghana survey for the Northern Region, no distinction was made of the time when non-campaign nets were lost, and this category is referred to as “pre-post campaign”.

For each lost net the reported age at the time of loss was recorded. The fate of the net was inquired and noted in 10 categories which in turn were grouped into two main categories: (i) nets given away for others to use (including those stolen) and (ii) those discarded. These categories follow the recommendation on assessment of causes for attrition established by WHO [5]. Subsequently, the reasons for the loss were explored.

Data were merged from the surveys and descriptive statistics, median ages, and regression analyses were conducted with Stata 12 (Stata Corporation, College Station, Texas, USA). All statistical analyses were done taking into account the design effect from the cluster survey design. Univariate logistic regression was performed with potential explanatory variables to determine the selection of variables for the multivariate regression. To assess differences in the distribution of net age at the time of loss for the different types of nets and country settings, inverse cumulative distributions were created and plotted against net age.

The wealth index was computed at the household level for each survey and strata using principal component analysis (PCA) [24]. The variables for household amenities, assets, livestock, and other characteristics that are related to a household’s socioeconomic status were used for the computation. All variables were dichotomized except those of animal ownership where the total number owned was used. The first component of the PCA was used as the wealth index. Households were then classified according to their index value into quintiles, calculated separately for each survey. For analysis of individual nets the quintile allocation of the household was applied.

Ethical clearance for the original post-campaign survey in Senegal was obtained from the Johns Hopkins University Bloomberg School of Public Health Institutional Review Board in Baltimore, Maryland, USA and the *Comité National d’Ethique pour la Recherche en Santé* in Dakar, Senegal. For Ghana, clearance was obtained from the Ghana Health Service Ethical Review Committee (Northern and Eastern), and from the JHSPH IRB (Eastern Region). In Nigeria, clearance was obtained from National Health Research Ethics Committee of Nigeria, and in Uganda, ethical review was provided by the Uganda National Council of Science and Technology (UNCST) in Kampala, Uganda.

## Results

### Global results

Among the 14 surveys a total of 14,196 households reported owning 25,447 nets of any kind (Table 1) on the day of the survey, of which 23,955 (94%) were LLINs. In addition, a total of 4,102 nets were reported to have left the households in the sample: of these, 2,580 were discarded (63%), and 1,383 were given away (34%). A small percentage (3.4%, or 155 nets) were reported lost but were missing fate (n = 139) or age (n = 16) (Table 1). These nets were excluded from the subsequent analysis, leaving 3,947 lost nets.

**Table 1:** **Number of nets in the sample**

	# of households	# of nets present on day of survey	# of nets that left the household	Total nets past and present
Uganda	549	1,646	397	**2,043**
Ghana	1,821	4,093	716	**4,809**
Senegal	1,540	7,029	1,818	**8,847**
Nigeria	10,286	12,679	1,171	**13,850**
Total	**14,196**	**25,447**	**4,102**	**29,549**
Lost nets with incomplete data			155	
Total lost nets for analysis			**3,947**	

Table 2 presents details of what happened to the lost nets. Of the nets reported discarded (2,465), 53% (1,298) were thrown away while 37% (921) were destroyed. Only 255 of discarded nets were reported used for other purposes, representing 6% of the total sample of lost nets (n = 3,947) and less than 1% of the total sample of nets owned by households (255/29,551).

**Table 2:** **Fate of nets lost from household**

	Freq.	Overall %	Within group %
Discarded	2,465	62.5%	
**Thrown away**	1,298	32.9%	52.7%
**Destroyed**	921	23.3%	37.4%
**Used for other purpose**	246	6.2%	10.0%
Given Away	1352	34.3%	
**Given or taken by relatives**	845	21.4%	62.5%
**Given to others**	420	10.6%	31.1%
**Stolen**	71	1.8%	5.3%
**Sold**	16	0.4%	1.2%
Unknown	130	3.3%	
**Do not know**	66	1.7%	50.8%
**Other**	64	1.6%	49.2%
Total	3,947	100	

Of the nets reported given away, the majority (845, or 62.5%) were given to or taken by relatives, while 31.1% (420) were given to non-relatives. Only 5.3% (71) were reported stolen, and 1.2% (16 nets out of the total sample) were reportedly sold.

### Age of nets when ‘lost’

**Figure 2:**
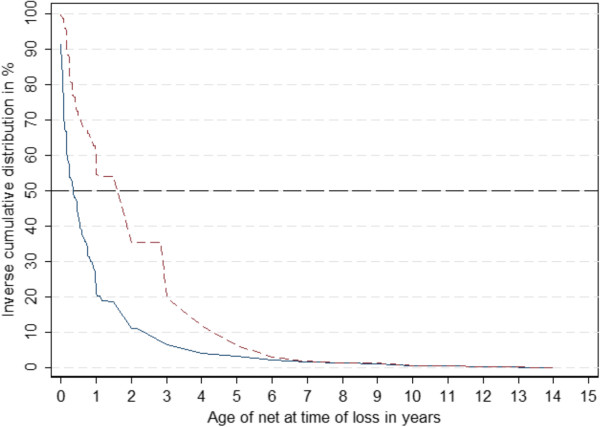
**Distribution of net age at time of loss for nets given away (solid blue line) and those discarded (dashed red line).**

**Figure 3:**
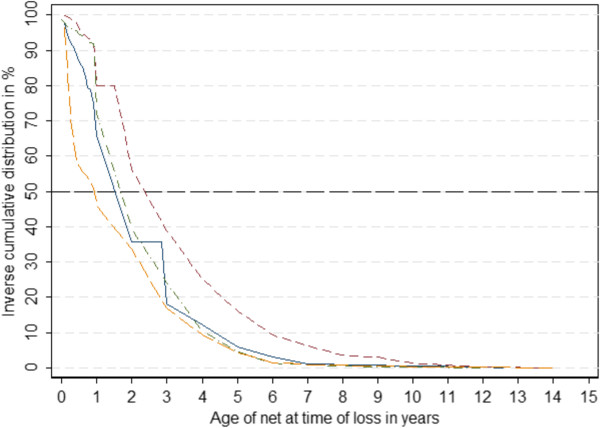
**Distribution of net age at time of loss by country.** Nigeria: solid blue line; Senegal: long-dash yellow line; Uganda: dash-dot green line; Ghana: dash red line.

The median age at which nets were reported lost was 0.96 years (Inter-Quartile Range [IQR] 0.25 – 3.00 years old). However, as shown in Figure 2, the age distribution differed significantly between nets given away to others and those discarded. Nets that were given away had a median age of 0.34 (IQR 0.08 – 1.00 years old), while nets that were discarded had a median age of 2.00 (IQR 0.42 – 3.00 years old). The difference between these groups was statistically significant (p = 0.0001, Kruskal-Wallis test). Nets given to other people were on average older than nets given to family members, at median ages of 0.62 (IQR 0.17 – 2.00) years old and 0.19 (IQR 0.06 – 0.77) years old, respectively and this difference was also statistically significant (p = 0.0001, Kruskal-Wallis test). No difference in age at loss was found for nets thrown away or destroyed versus those used for other purposes (p > 0.05, Kruskal-Wallis test).Distribution of age at time of loss also differed by country (Figure 3). In Senegal, more nets were lost within the first year compared to the other countries. In contrast, loss of nets occurred generally later in Ghana while the age distribution curves of lost nets were very similar in Nigeria and Uganda.

### Determinants of fate of ‘lost’ nets in univariate analysis

Households dealt differently with nets obtained from campaigns and nets obtained through other sources: campaign nets represented 73.0% of nets given away, compared to non-campaign nets (21.4%, p < 0.0001). The other major determinant was the age of net at time of loss: 51.4% of nets less than 6 months old were given away, 44.1% of those between 6 months and one year, 24.2% aged 1–3 years and 13.9% of nets older than three years (p < 0.0001). Table 3 combines these two determinants and shows that for campaign nets, most of the “giving away” occurred within the first months after distribution. Non-campaign nets also showed a declining trend but the proportion given away rather than discarded was much lower throughout.

**Table 3:** **Proportion of previously owned nets given away by type of net and age at loss**

Type of net	Reported age of net at time of loss in months							Total
	0-1	1-3	3-6	6-12	12-36*	36-60	>60	
Campaign								
N	379	200	155	200	51			985
% of nets given away (vs discarded)	87.6%	66.5%	62.6%	62.5%	62.8%	No data	No data	73.0%
95% CI	80.2 – 92.3	56.1 – 75.5	47.4 – 75.6	50.4 – 73.2	45.1 – 77.5			67.5 – 77.9
Non-campaign								
N		328	420	294	862	694	364	2962
% of nets given away (vs discarded)	No data	35.2%	20.0%	33.0%	21.9%	13.4%	14.8%	21.4%
95% CI		25.8 – 45.9	13.9 – 28.0	24.5 – 42.8	18.1 – 26.4	10.6 – 16.8	10.5 – 20.5	18.7 – 24.3

*for campaign nets only up to 16 months maximum.

A number of other factors were identified in the univariate analysis that showed a significant association with whether a net was given away for others to use rather than discarded. The proportion given away was higher in urban settings (38.7% vs. 30.3% rural, p = 0.007), in smaller sized households (52.1% if 1–3 people, 38.4% for 4–6 people and 26.3% for 7 or more, p < 0.0001), among households with more educated heads of household (29.9% if non-literate, 34.0% if primary education, 38.4% if secondary and 40.1% if tertiary, p = 0.02), and among households that did not have any children under 5 (41.4% vs. 29.7% with children, p < 0.0001). The proportion of lost nets given away also differed by country with Nigeria showing the highest rate (49.2%), followed by Uganda (39.6%), Ghana (29.4%) and Senegal (24.2%, p < 0.0001).

Household socio-economic status (wealth quintiles) was not statistically associated with the fate of the lost nets in the univariate analysis, nor was there a difference between non-campaign nets lost before or after the campaign or between households that owned enough nets for all members (1 net for 2 people) and those that did not.

### Multivariate logistic regression

Since a number of the variables found to be associated with giving nets away in the univariate analysis are inter-related, such as household size and presence of children under five, and others significantly differed between countries, such as educational status, a multivariate logistic regression was used to assess the determinants of a previously owned net being given away. Results are shown in Table 4 and confirm that campaign nets were almost six times (OR 5.95, 4.25-8.32, p < 0.0001) more likely to be given away than non-campaign nets lost during the same period between campaign and survey. The regression model also revealed that non-campaign nets lost before the campaign were significantly less likely to be given away compared to non-campaign nets lost after the campaign (OR 0.57, 0.39-0.83, p = 0.004). In contrast to the univariate analysis the model also suggests that households are more likely to give away nets if they have more nets than one for every two household members (OR 1.88, 1.37-2.54, p < 0.0001). Other factors that were confirmed to be significantly associated with giving away the net were age of net at time of loss, urban residence and country.

**Table 4:** **Multi-variable logistic regression models of determinants of giving lost net away to others**

Explanatory variables	Adjusted odds ratio	95% CI	P value
Type of net and period of loss			
Non-campaign net lost post-campaign	1.00		
Campaign net lost post-campaign	5.95	4.25 – 8.32	< 0.0001
Non-campaign net lost pre-campaign	0.57	0.39 – 0.83	0.004
Non-campaign net lost pre-post campaign	1.45	0.70 – 3.00	0.31
LLIN owned by the household			
Less than 1 LLIN/2 people	1.00		
Exactly 1 LLIN/2 people	1.29	0.92 – 1.80	0.14
More than 1 LLIN/2 people	1.88	1.39 – 2.54	< 0.0001
Age of net at time of loss			
0-5 months	1.00		
6-11 months	0.75	0.52 – 1.09	0.13
12-35 month	0.47	0.32 – 0.69	< 0.0001
36+ months	0.32	0.22 – 0.47	< 0.0001
Country			
Nigeria	1.00		
Ghana	0.45	0.25 – 0.83	0.01
Uganda	0.79	0.45 – 1.40	0.42
Senegal	0.24	0.15 – 0.37	< 0.0001
Rural	1.00		
Urban	1.79	1.27 – 2.53	0.001
Number of household members			
1-3 people	1.00		
4-6 people	0.82	0.59 – 1.15	0.24
7 or more people	0.70	0.49 – 0.98	0.045
Wealth quintile			
Wealthiest	1.00		
Fourth	0.91	0.63 – 1.31	0.60
Middle	0.95	0.61 – 1.47	0.81
Second	0.97	0.62 – 1.51	0.88
Poorest	0.62	0.38 – 1.02	0.06
Educational level of head of household			
Tertiary	1.00		
Secondary	1.03	0.62 – 1.71	0.90
Primary	1.01	0.62 – 1.64	0.99
Non-literate	1.36	0.83 – 2.22	0.22
Number of children under five			
None	1.00		
Any	0.97	0.74 – 1.27	0.85

In contrast, educational status of the head of household or having children under five were no longer influential after controlling for other factors, and the association with household size was much weaker than in the univariate analysis, with only large households showing a statistically significant reduction in the probability of giving away a net compared to small families.

**Figure 4:**
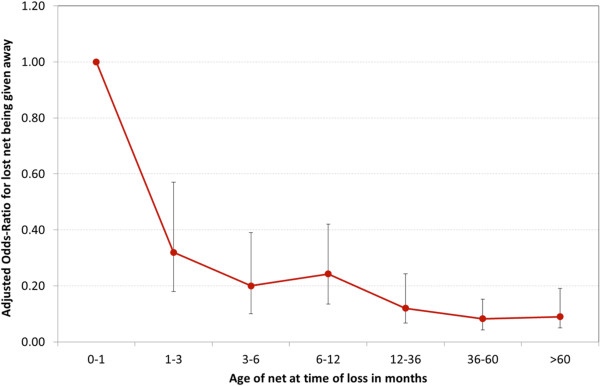
**Odds-Ratio of a net being given away as a function of time.** Adjusted OR based on model presented in Table 4.

The adjusted odds ratios for all previously owned nets being given away by age at the time of loss are presented in Figure 4 using the covariates as shown in Table 4 but with a more detailed breakdown of age of net intervals. This demonstrates that the most likely period a net was given away to others was within a month of obtaining it. Even for nets one to three months old at time of loss the odds of giving it away were less than half compared to a new net and then continuously declined to reach about one tenth of the odds ratio after more than one year.

### Nets used for other purposes

Overall 6.2% of previously owned nets were reported to have been used for purposes other than sleeping under, but rates significantly varied between countries (p < 0.0001). Senegal had the highest rate, at 11.1% of lost nets (201 of 1,818 nets), followed by Uganda at 4.0% (16 of 397 nets), and Nigeria at 2.1% (25 of 1,171 nets) while Ghana had the lowest rate, at 1.8% (13 of 716 nets). Relating the nets used for other purposes to the overall total of all nets found in the surveyed households (see Table 2) gives an estimate of misuse of nets of only 0.9% (255 of 29,549 nets).

### Reasons for loss of nets

Respondents were asked to provide the reason for the loss of the net. The vast majority of nets that were destroyed or thrown away were described as “too torn” (86% and 93%, respectively). Of nets that were given to others, 71% of respondents said it was because the net was not needed. For the 19 nets that were sold, seven were sold because the household needed money, seven because they were not needed, and four were reportedly sold because they were too torn. Three quarters of the nets that were used for other purposes were described as too torn. Even for the few (148) nets that were relatively new (under a year old) and used for another purpose, 66% were used for other purposes because the net was reportedly too torn.

## Discussion

Campaign nets and non-campaign nets were treated differently by households. Campaign nets were nearly six times more likely to be given away than non-campaign nets, and nets were far more likely to be given away in the first month following a campaign, suggesting that the bulk of redistribution among family and friends occurs in this period. Non-campaign nets, which were generally older and may not have been LLINs, were more likely to have been discarded, most likely being replaced by newer nets acquired through the mass campaign.

Discarding nets was primarily associated with the age and condition of the net – nets were discarded because they were too torn, and at a median age of two years. This does not however indicate that the median lifespan of nets is two years. Median lifespan cannot be calculated solely from observing remaining nets, or on the basis of the age of discarded nets. It is calculated by dividing the number of LLIN originally received, minus those given away, by the number of current LLIN present in the household that are in serviceable condition, as follows:

The criteria of being serviceable is based on the proportionate Hole Index [5] result for each net using a cut-off that is equivalent to a total estimate hole surface of the net of more than 0.1 square meters [4].

The multivariate regression provides insights into determinants of the fate of nets, either given away or discarded. None of the findings are surprising; households that have more nets than they need are more likely to give away nets, as are urban households, who may be in closer proximity to relatives or friends in need of nets. It is worth emphasizing that level of education and wealth quintile did not significantly affect net fate.

Differences by country are apparent but inscrutable: the relatively younger age of discarded nets in Senegal could potentially reflect harsher conditions, more consistent usage leading to net wear and tear, or socio-cultural factors. As an example of the latter, recent qualitative research in Nigeria, Senegal, and Uganda indicates that households appear to value the look of an intact net as a reflection on the cleanliness and housekeeping skills of the family [25, 26] (Scandurra, in preparation). Qualitative research conducted in the Dadaab refugee camps in Kenya also revealed that having only a few holes in one’s net was considered cause for discarding it [27], and other studies found that net usage was the main contributor to holes in nets [7, 10]. A combination of increased use and stress on the net with social norms making torn nets less desirable could contribute to discarding of nets at younger ages when they still have a limited number of holes. On the other end of the spectrum, nets that are retained for longer periods of time may be due to households saving nets without using them for some time prior to hanging them up, effectively postponing use of (and wear and tear on) the net, leading to an older crop of nets in better condition. That household decision-making on net end of life is highly subjective creates an opportunity for behaviour change communication activities to contribute to promoting keeping nets for longer periods of time, keeping them in better condition through preventive actions such as tying nets up during the day, and even repair behaviours, discussed further in two forthcoming studies.

This analysis shows that redistribution of LLINs does occur following mass campaigns, primarily to family members, but also to non-family members. The scale of this redistribution is small but important: recent continuous distribution pilots of school-based distribution to children in selected classes operate on an assumption that households that receive an excess of LLINs for their needs will give extra nets away to community members that did not benefit from the distribution, or who need additional nets [15]. The overall rate of net redistribution in this sample was just under 5% of all nets (1,383 nets out of 29,549 nets owned by households).

Open-ended answer options in these datasets indicated that nets are given in a large number of cases to students in school (particularly boarding school) or given to or taken by family members residing in other areas. This is also the case in a separate study in Uganda (Helinski, personal communication). Further research is needed to assess under what circumstances households would preferentially hoard or give away nets, and to whom. Since it already occurs to a limited degree, LLIN redistribution may be a behaviour that can be encouraged as part of distribution channels that reach only a selected target population, such as school distributions that target selected classes on a yearly basis. These channels miss households that have no school-aged children, although these households do not make up a large proportion of the overall population [15]. These data also suggest that the conditions under which net redistribution is more likely to occur are net-rich environments, where population access to LLIN is relatively high. Continuous distribution through schools may have an opportunity to build up LLIN access within households over time. Given that LLIN are more likely to be redistributed soon after a distribution, school distributions could promote this behaviour as part of their targeted messaging to parents and school-children, to capitalize on the brief window of opportunity within the first month or two post-distribution.

There are limited studies on the use of nets for purposes other than sleeping under [16], and observational studies are generally limited to a particular study area [10, 17, 20]. This analysis makes an important contribution to the quantification of this perceived problem, which tends to be exaggerated or exacerbated by newspaper reports of nets being used for protecting crops or as soccer goals. The evidence here clearly demonstrates that in general, across several geographic areas and time points, use of nets for other purposes is very rare, at less than 1% of nets, and that when it occurs, it happens primarily with older nets. National malaria programmes, ministries of health, donors, and implementing agencies should, therefore, remain confident that their investments in malaria control are being used effectively. As Eisele *et al.* have also shown, it is unlikely that the use of old or no-longer-needed nets for other purposes is impeding the use of nets for malaria prevention within households [16], rather, households are reusing materials once they are deemed no longer useful for sleeping under, or because they are an extra net that is not needed. Echoing the present findings, in coastal Kenya, Mutuku *et al.* found that between 60-80% of nets used for chicken shelter, window screening, fencing and other purposes were over two years old [10]. Certainly, in specific areas, the economic benefits of using nets for fishing outweigh the perceived health benefits of using the nets for sleeping, and this phenomenon has even been described using econometric game theory models in Honjo *et al.*[21]. Given that net misuse of this type is rational economic behaviour, this requires action not only to ensure that residents of that area are protected from malaria, but also to improve households’ economic status, to diminish the marginal utility of misusing nets. From an environmental standpoint, it is important to prevent overfishing of fish fry, endangering the food supply, and to prevent pyrethroids from leaching into water systems, where they can be toxic to a large range of aquatic life [28–30].

Taken together, these data from this study indicate a relatively consistent pattern and distribution of nets given away *vs* discarded (Figure 3). Studies that do not quantify the number of nets given away or discarded in their durability calculations could use data from the present study to adjust their estimates. In the absence of information regarding the rates at which nets are lost, and the reasons for their loss, these data could be used in retrospective durability studies to adjust for recall bias. Retrospective durability studies rely on the recall of household members on when they received their nets, from which source, and what happened to nets lost from the household.

### Limitations

As the data in this analysis come from retrospective cross-sectional surveys, it is possible that details about the nets were affected by recall bias, particularly for the age of the nets acquired through channels other than a recent mass campaign. It is also possible that respondents may have underreported repurposing of nets to the enumerators; to reduce this response bias, the questionnaire was structured to ask the fate of each lost net as an unprompted question. While LLINs from Nigeria appear overrepresented in the total sample, each state in Nigeria conducted its own campaign and has unique sociocultural and environmental factors, providing rich and diverse data even within a single country.

## Conclusions

This analysis has shown that inter-household re-allocation of nets does occur, but was sensitive to current household net ownership and the time elapsed since mass distribution. These results have important implications for continuous LLIN distribution strategies, which have to date assumed that redistribution of excess nets between households occurs without any supporting or contradictory evidence. These factors can be addressed programmatically to further facilitate reallocation within a given community. Continuous distribution channels that rely in part on reallocation to achieve broad community coverage will need to focus on BCC and other activities to encourage this practice.

A second important finding was that the overwhelming majority of nets were used for malaria prevention. Of the repurposed nets (<1% overall), the majority were already considered too torn, indicating they had already served out their useful life for malaria prevention. National programmes and donor agencies should remain confident that overall, their investments in LLIN are being appropriately used.
